# Correlation Between Arrhythmia and the Prognosis in Children With EFE/LVNC/DCM

**DOI:** 10.3389/fped.2020.00280

**Published:** 2020-06-10

**Authors:** Hong Wang, Yan-Qiu Chu, Xian-Yi Yu, Rui Chen, Yan-Lin Xing, Xue-Xin Yu, Ce Wang, Le Sun, Yun-Ming Xu, Xue-Mei Li, Xiao-Zhe Cui

**Affiliations:** Department of Pediatrics, Shengjing Hospital, China Medical University, Shenyang, China

**Keywords:** children, Wolff–Parkinson–White syndrome, cardiomyopathy, arrhythmia, prognosis, LVEF

## Abstract

**Aim:** To explore the correlation between different phenotypes of arrhythmia and the prognosis in children with EFE/LVNC/DCM.

**Methods:** A total of 167 children with cardiomyopathy diagnosed and treated in Shengjing Hospital between January 2010 and May 2019 were evaluated. After patient screening, 31 patients with endomyocardial fibroelastosis (EFE), left ventricular non-compaction, or dilated cardiomyopathy with significant arrhythmias were selected. In addition, 42 children with primary EFE were selected to evaluate the prognosis with or without arrhythmia. Follow-up was undertaken 0, 1, 3, 6, 9, and 12 months after treatment.

**Results:** We revealed the outcomes for five types of cardiomyopathy: EFE patients with Wolff–Parkinson–White syndrome B and supraventricular tachycardia, intraventricular block and complete left bundle branch block recovered slower than EFE patients with atrial flutter and atrial fibrillation, even slower than EFE with ventricular tachycardia. The average recovering time for LVEF and LVED in EFE patients without arrythmia was 10 months after diagnosis, while 76.9% (3/13 cases) of those with significant arrythmia hadn't recovered until 24 months after diagnosis. Three of patients died at 6, 7, and 6 and half years after diagnosis.

**Conclusion:** The long-term prognosis in children with cardiomyopathy is associated with the type of arrhythmia and time of intervention. The prognosis of EFE patients with arrhythmia is worse than EFE patients without arrhythmia. Patients with Wolff–Parkinson–White syndrome B, especially a significantly widen QRS complex, carry a poor prognosis if radiofrequency ablation is not undertaken. CLBBB patients have similar poor prognosis if proper pacemaker is not implanted timely.

## Background

Endomyocardial fibroelastosis (EFE), left ventricular non-compaction (LVNC), and dilated cardiomyopathy (DCM) are common phenotypes of cardiomyopathy in children. Usually, in these patients with non-ischemic cardiomyopathy, scar progression contributes to increased left ventricular end-diastolic diameter (LVED), left ventricular ejection fraction (LVEF), and ventricular tachycardia (VT) (1). The prognosis of pediatric patients with cardiomyopathy is correlated with arrhythmias. EFE, LVNC, and DCM in children, are usually accompanied with supraventricular tachycardia (SVT), atrial fibrillation (Af), atrial flutter (AF), VT, frequent premature ventricular contraction (PVC) and even involvement of the conduction system [e.g., inter-ventricular conduction block (IVB) and complete left bundle branch block (CLBBB)]. Most of these patients are diagnosed at <1 year of age (2).

In recent years, clinicians are more aware of the association of patient prognosis with phenotypes of cardiomyopathy, cardiac function, and age of the diagnosis. Though nearly one-quarter of pediatric patients with cardiomyopathy have arrhythmia in Shengjing Hospital at China Medical University (Shenyang, China), we had not studied the relationship between the prognosis and arrhythmia until the previous 5 years. The prognosis can be very different based on the type of arrhythmia a baby/infant has.

Cardiomyopathy with the Wolff–Parkinson–White syndrome (WPW) in adults was first reported in 1982 (3). Until 1997, the dangers of WPW in children had not been recognized (4). There have been increased number of studies focusing on treatment of WPW (5) and CLBBB (6) with cardiac insufficiency (7).

## Methods

We have received the Ethical approval of the study protocol.

Based on the type of arrythmia, total 31 EFE/LVNC/DCM patients with arrythmia were divided in to 3 group, group 1 (*n* = 9): with WPW-B (*n* = 3)/IVB (*n* = 3)/CLBBB (*n* = 3); group 2 (*n* = 11): with AF/Af/AT; group 3 (*n* = 11): with PVC/VT. In addition, total 42 primary EFE patients were divided into A and B groups. Group A (*n* = 13): EFE with arrythmia; group B (*n* = 29): EFE without arrythmia.

### Inclusion Criteria

The criteria of EFE/LVNC/DCM diagnosis was according to lecterture reports (8–10). The significant arrhythmia included Af, AF, AT, SVT, IVB/CLBBB, PVC, and VT, and WPW-B with widen QRS complex [QRS duration > 120 ms, assessed using leads V_5_ or II on standard 12-lead electrocardiography (ECG)].

### Exclusion Criteria

**A**rrhythmia-induced right ventricular cardiomyopathy (ARVC); hypertrophic cardiomyopathy (HCM); restrictive cardiomyopathy (RCM); incomplete right bundle branch block (IRBBB) or complete right bundle branch block (CRBBB); channelopathies such as Brugada, long QT syndrome, atecholaminergic polymorphic ventricular tachycardia.

### Data Collection

Data on sex, age, cardiac markers including cardiac troponin I (cTnI), high sensitivity cardiac troponin T (hs-cTnT), and N-terminal pro brain natriuretic peptide (NT pro-BNP), LVEF, and changes in LVED (ΔLVED) at the initial diagnosis (0) as well as at 1, 3, 6, 9, and 12 months after treatment were obtained.

### Analyses

Data *t*-test was used for statistic significance (*p* < 0.05). Two-way ANOVA was used, too. By Kaplan–Meier analysis, the outcomes at 36 months among Group 1, 2, and 3 patients, and between Group A and B were counted as persistent LVEF of ≤ 35% or for event (11).

## Results

### General Conditions Before Treatment

The value of ΔLVED in Group 3 (with PVC/VT) was significantly lower than that in Group 1 (WPW-B/IVB/CLBBB) (*p* < 0.01–0.05). Based on NYHA the chronic heart failure was worse in EFE with arrythmia (Group A) than without it (Group B). Levels of cTnI, hs-cTnT, and NT pro-BNP were not significantly different in different groups before treatment.

### Dynamic Changes in the cTnI Level

The cTnI level in each group was higher before treatment than after one, while the cTnI level in Group 2 was higher than Group 1 and 3, the differences were not significant (Table 1).

**Table 1:** The dynamic changes of cTnI.

**Group**	***n***	**Age**	**cTnI-0M**	**cTnI-1M**	**cTnI-3M**	**cTnI-6M**	**cTnI-9M**	**cTnI-12M**
1	9	1.4 ± 1.2	0.098 ± 0.095	0.089 ± 0.147	0.029 ± 0.024	0.020 ± 0.020	0.043 ± 0.046	0.028 ± 0.031
2	11	2.9 ± 4.0	0.167 ± 0.117	0.014 ± 0.006	0.028 ± 0.027	0.092 ± 0.161	0.029 ± 0.038	0.010 ± 0.000
3	11	2.6 ± 4.0	0.056 ± 0.057	0.019 ± 0.010	0.012 ± 0.005	0.017 ± 0.011	0.012 ± 0.003	0.008 ± 0.004
A	13	0.8 ± 0.7	0.265 ± 0.486	0.079 ± 0.121	0.028 ± 0.023	0.011 ± 0.002	0.023 ± 0.029	0.022 ± 0.026
B	29	0.7 ± 0.6	0.303 ± 0.431	0.026 ± 0.021	0.015 ± 0.009	0.015 ± 0.014	0.014 ± 0.009	0.010 ± 0.000

*Group 1, WPW-B+SVT/IVB/CLBBB; Group 2, AF/Af/AT; Group 3, PVC/VT; Group A, EFE with arrythmia; Group B, EFE without arrythmia*.

### Dynamic Changes in the hs-cTnT Level

The hs-cTnT level in each group was higher before treatment than after one, but the difference was not significant. The hs-cTnT level in Group 1 was lower than Group 2 and 3, but both the differences were not significant. Two way ANOVA showed there are significant difference between NYHA in Group 1, 2, and 3 at hs-cTnT-5 point (*p* < 0.01) (Table 2).

**Table 2:** The dynamic changes of hs-cTnT.

**Group**	**Hs-cTnT-0M**	**Hs-cTnT-1M**	**Hs-cTnT-3M**	**Hs-cTnT-6M**	**Hs-cTnT-9M**	**Hs-cTnT-12M**	**NYHA-0M**
1	0.058 ± 0.036	0.037 ± 0.041	0.023 ± 0.024	0.027 ± 0.021	0.029 ± 0.019	0.013 ± 0.007	3.9 ± 0.3
2	0.104 ± 0.099	0.020 ± 0.020	0.028 ± 0.017	0.056 ± 0.073	0.028 ± 0.030	0.008 ± 0.000	3.6 ± 0.5
3	0.100 ± 0.129	0.021 ± 0.008	0.020 ± 0.011	0.022 ± 0.020	0.020 ± 0.014	0.006 ± 0.003	3.7 ± 0.5
A	0.051 ± 0.040	0.040 ± 0.031	0.022 ± 0.018	0.024 ± 0.019	0.019 ± 0.016	0.011 ± 0.007	3.9 ± 0.3
B	0.092 ± 0.122	0.022 ± 0.017	0.014 ± 0.012	0.019 ± 0.021	0.013 ± 0.011	0.006 ± 0.003	3.5 ± 0.6

*Group 1, WPW-B+SVT/IVB/CLBBB; Group 2, AF/Af/AT; Group 3, PVC/VT; Group A, EFE with arrythmia; Group B, EFE without arrythmia*.

### Dynamic Changes in the NT Pro-BNT Level

The NT pro-BNP level was significantly lower at any points after treatment than that of before in Group 2 (*p* < 0.05–0.01). In group 1, the NT pro-BNP level was lower at any point after treatment, except at 1 year, than that of before, had significant difference (*P* < 0.05–0.01). In group 3, only after 1 year treatment, the NT pro-BNP level was higher than that of before, had significant difference (*P* < 0.05). Two ways ANOVA showed there are significant difference between NYHA in 1, 2, and 3 groups at NT pro-BNP-1 (*P* = 0.02) (Table 3).

**Table 3:** The dynamic changes of NT pro-BNP.

**Group**	**NT pro-BNP−0M**	**NT pro-BNP−1M**	**NT pro-BNP−3M**	**NT pro-BNP−6M**	**NT pro-BNP−9M**	**NT pro-BNP−12M**	**NYHA-1**
1	18,061 ± 15,480	2,209 ± 1,428^a^	1,904 ± 1,768^a^	2,070 ± 2,027^a^	251 ± 69^a^	6,816 ± 11,640	3.9 ± 0.3
2	23,692 ± 14,706	5,037 ± 6,810^b^	3,393 ± 4,472^b^	3,027 ± 4,476^b^	2,952 ± 5,398^b^	179 ± 118^b^	3.6 ± 0.5
3	10,342 ± 13,226	2,198 ± 2,096	1,417 ± 1,465	1,180 ± 1,495	1,053 ± 1,304	234 ± 233^a^	3.7 ± 0.5
A	18,771 ± 15,335	4,283 ± 5,876	2,603 ± 3,384	1,632 ± 1,733	369 ± 446	3,134 ± 7,938	3.9 ± 0.3
B	19,026 ± 13,359	3,136 ± 2,621	1,241 ± 1,404	740 ± 1,315	298 ± 374	294 ± 438	3.5 ± 0.6

Group 1, WPW-B+SVT/IVB/CLBBB; Group 2, AF/Af/AT; Group 3, PVC/VT; Group A, EFE with arrythmia; Group B, EFE without arrythmia.

a p < 0.05, compared to NT pro-BNP-0M;

b *p < 0.01 compared to NT pro-BNP-0M*.

### Dynamic Changes in LVEF

LVEF returned to persistent normal in Group 2 and 3 at 12 months after treatment significantly (*p* < 0.05–0.01). However, in Group 1, LVEF did not return to normal until 1 year after treatment. At 6, 9, and 12 months after treatment, LVEF in Group 2 and 3 recovered significantly faster than in Group 1 (*p* < 0.05–0.01). LVEF recovered significantly faster in Group B after treatment at any point compared to before treatment (*p* < 0.05–0.01). Two-way ANOVA showed there were significant differences between types of arrythmia in Group 1, 2, and 3 at LVEF-6M (*p* < 0.01) and between NYHA in Group 1, 2, and 3 at LVEF-1M (*p* < 0.01) (Table 4).

**Table 4:** The dynamic changes of LVEF.

**Group**	**LVEF-0M**	**LVEF-1M**	**LVEF-3M**	**LVEF-6M**	**LVEF-9M**	**LVEF-12M**	**NYHA-0M**
1	32.1 ± 8.6	42.8 ± 13.9	42.5 ± 18.1	32.6 ± 4.8	36.5 ± 8.3	35.2 ± 9.0	3.9 ± 0.3
2	37.1 ± 11.7	48.3 ± 13.7	56.6 ± 12.8^h^	54.1 ± 13.6^b^^,^ ^g^	52.9 ± 14.9^a^^,^ ^g^	57.6 ± 7.2^b^^,^ ^h^	3.6 ± 0.5
3	30.6 ± 7.5	37.5 ± 9.1	46.8 ± 14.7^g^	43.7 ± 8.9^a^^,^ ^h^	50.0 ± 9.1^a^^,^ ^h^	57.5 ± 23.6^b^^,^ ^h^	3.7 ± 0.5
A	34.8 ± 8.6	41.2 ± 12.1	44.5 ± 18.4	40.2 ± 13.4	41.5 ± 8.8	42.2 ± 11.2	3.9 ± 0.3
B	33.1 ± 10.7	39.5 ± 9.2^e^	44.8 ± 13.3^f^	47.8 ± 12.7^f^	50.6 ± 11.7^c^^,^ ^f^	56.1 ± 10.2^d^	3.5 ± 0.6^b^^,^ ^f^

Group 1, WPW-B+SVT/IVS/CLBBB; Group 2, AF/Af/AT; Group 3, PVC/VT; Group A, EFE with arrythmia; Group B, EFE without arrythmia.

a p < 0.05, compared to Group 1;

b p < 0.01, compared to Group 1;

c p < 0.05, compared to Group A;

d p < 0.01, compared to group A;

e p < 0.05, compared to Group A;

f p < 0.01, compared Group A;

g p < 0.05, compared to LVEF-0M;

h *p < 0.01 compared to LVEF-0M*.

### Dynamic Changes in ΔLVED

After half year of treatment, LVED was recovered significantly faster in Group 2 and 3 than that in Group 1 (*p* < 0.05–0.01). After 1 year of treatment, LVED was almost recovered in Group 2 and 3, but was worsened in Group 1. Two-way ANOVA showed there were significant difference between the types of arrythmia in Group 1, 2, and 3 at LVED-6M (*p* < 0.01) and LVED-12M (*p* < 0.01) (Table 5).

**Table 5:** The dynamic changes of ΔLVED.

**Group**	**ΔLVED-0M**	**ΔLVED-1M**	**ΔLVED-3M**	**ΔLVED-6M**	**ΔLVED-9M**	**ΔLVED-12M**
1	15.2 ± 6.4	15.7 ± 6.7	12.3 ± 5.4	21.8 ± 5.6	19.8 ± 6.2	18.0 ± 4.0
2	12.9 ± 12.2	11.4 ± 10.7	6.1 ± 4.4^a^	8.6 ± 9.6^b^	7.4 ± 9.4^a^	2.9 ± 2.9^b^^,^ ^d^
3	12.7 ± 6.8	12.1 ± 6.9	10.7 ± 6.1	7.9 ± 5.3^b^	6.9 ± 5.4^a^	4.4 ± 4.2^b^^,^ ^e^
A	12.5 ± 4.7	11.4 ± 6.0	10.5 ± 6.2	14.6 ± 9.3	14.4 ± 7.5	12.4 ± 7.4
B	14.7 ± 7.2	12.8 ± 7.5	9.1 ± 7.4	8.1 ± 11.1	6.3 ± 6.5^c^	4.6 ± 6.0^c^

Group 1, WPW-B+SVT/IVB/CLBBB; Group 2, AF/Af/AT; Group 3, PVC/VT; Group A, EFE with arrythmia; Group B, EFE without arrythmia.

a p < 0.05, compared to Group 1;

b p < 0.01, compared to Group 1;

c p < 0.05, Compared to Group A;

d p < 0.05 and

e *p < 0.01, compared to ΔLVED-0M*.

### Trends in Myocardial Markers, LVEF and ΔLVED

**Figure 1:**
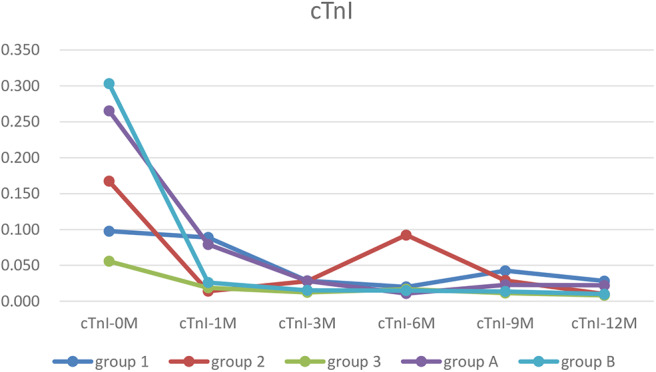
The trends of cTnI. In each group, cTnI was higher before treatment than after. It was recovered in all groups at 12 months of treatment point (Group 1, WPW-B+SVT/IVB/CLBBB; Group 2, AF/Af/AT; Group 3, PVC/VT; Group A, EFE with arrythmia; Group B, EFE without arrythmia).

**Figure 2:**
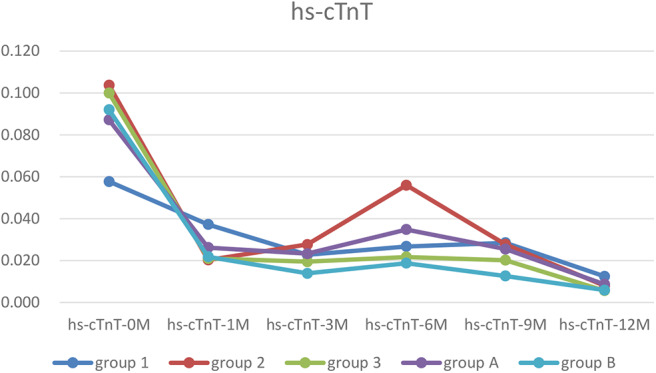
The trends of hs-cTnT. In each group, the hs-cTnT level was higher before treatment, and was almost recovered after 12 months treatment.

**Figure 3:**
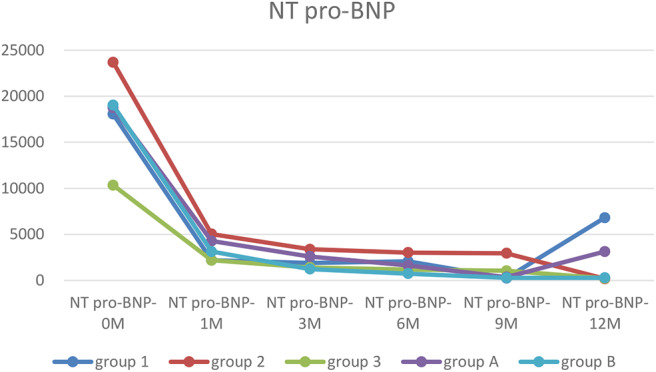
The trends of NT pro-BNP. Before treatment, NT pro-BNP were significantly elevated in every group. After treatment, cardiac functions were improved, and NT pro-BNP was quickly decreased and remained at a low level except in Group 1.

**Figure 4:**
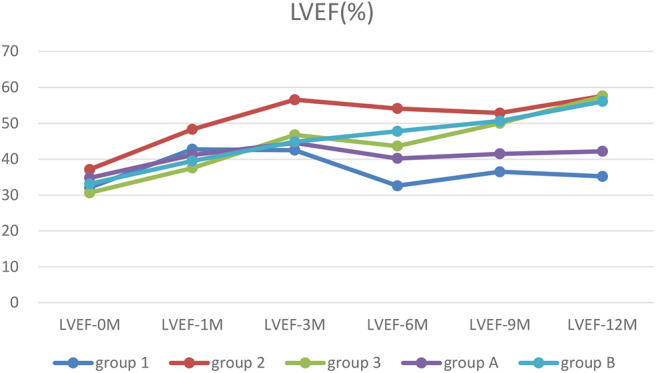
The trends of LVEF. Before treatment, all patients had low LVEF. After treatment, LVEF in patients with ventricular unsynchronization in Group 1 and A remained at a low level.

**Figure 5:**
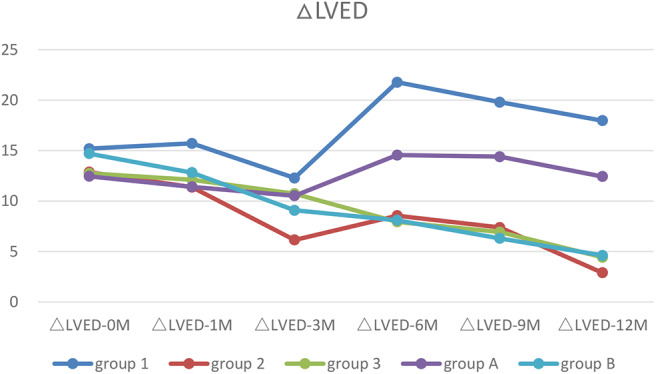
The trends of ΔLVED. Before treatment, all patients with increased LVED (ΔLVED). After treatment, only patients with ventricular unsynchronization in Group 1 and A had higher ΔLVED.

**Figure 6:**
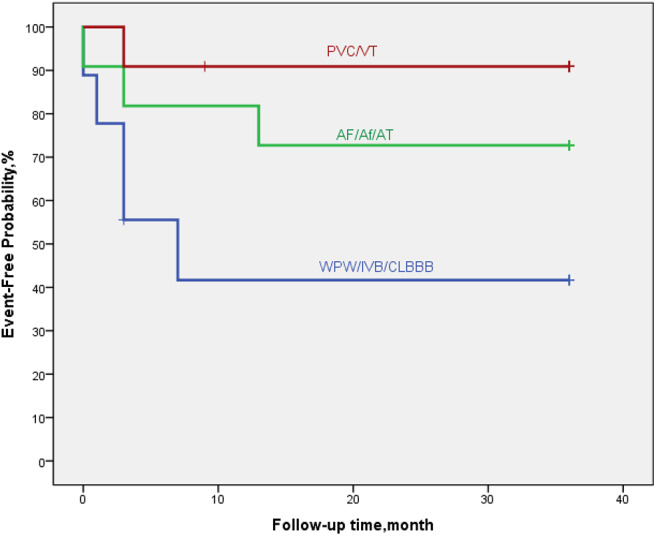
By Kaplan–Meier analysis, EFE/LVNC/DCM patients with arrythmia of WPW-B/IVB/CLBBB showed a high risk of poor outcome. The hazard ratio for persistent LVEF of ≤35% or for event was 7.0 (95% CI, 5.6–28.9; *p* = 0.052).

**Figure 7:**
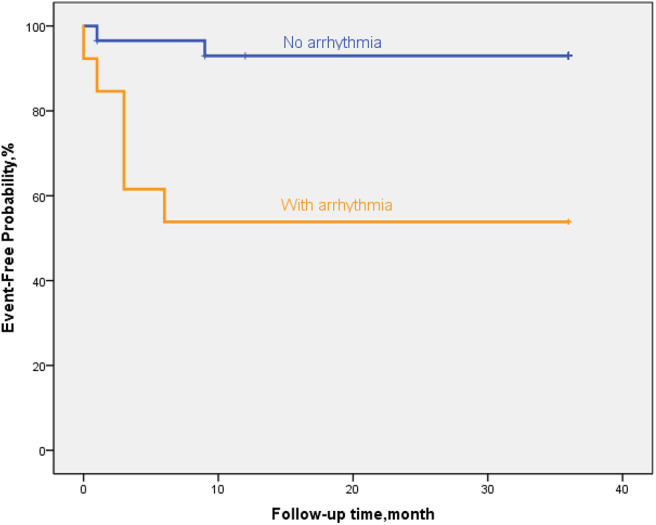
By Kaplan–Meier analysis, EFE patients with arrythmia showed a high risk of poor outcome. The hazard ratio for persistent LVEF of ≤35% or for event was 20.6 (95% CI, 11.6–29.7; *p* = 0.002).

Myocardial markers including cTnI, hs-cTnT, NT pro-BNP were analyzed before and after treatment (Figures 1–3). In addition, cardiac functions including LVEF and changes in LVED were analyzed (Figures 4, 5). Results are summarized in the following figures. The hazard ratio for persistent LVEF of ≤ 35% or for event between different groups are analyzed using Kaplan–Meier analysis (Figures 6, 7).

## Discussion

EFE, LVNC, and DCM are common types of cardiomyopathy in children, whereas HCM, arrhythmia-induced right ventricular cardiomyopathy (RVAC), and RCM are not (12). The prevalence of DCM in children younger than 1 year old is significantly higher than that in people 1–18 years old. It is higher in boys than in girls, and higher in non-Caucasians than in Caucasians (2). Although the incidence of RCM is low, the survival rate in children is low unless heart transplantation is carried out (12). RVAC is a hereditary cardiomyopathy that was indicated first in the 1980s. It is the most common cause of sudden death in adolescents, though far less common than EFE and LVNC (13).

We studied the correlation between the phenotype of cardiomyopathy associated with arrhythmia and left-ventricular systolic function in children even though LVED/LVEF was normal in some patients with HCM or RCM. RVAC shows involvement of the left ventricle in some patients (14), involvement of the right ventricle in some cases, and double-ventricular involvement in other people (15). So in this study, HCM/RCM, and RVAC were excluded.

There are often no clinical symptoms, and rarely altered cardiac function in children who underwent surgery at birth or who had surgery after birth that was complicated with CRBBB. In several studies on chronic and acute coronary artery disease, LBBB has been found to be an excellent predictor of mortality and events (16, 17). Arrhythmia in children manifests mainly as repeated SVT, atrial tachycardia, and VT. Unlike adults, infants develop atrial arrhythmias or SVT, and it is more persistent, often presenting with endless SVT or atrial tachycardia (18). However, these disorders mostly resolve before 1 year of age, and disordered atrial tachycardia usually resolves within 4 months (19). The long-term prognosis is good, as long as arrhythmias are controlled appropriately during this period.

**Figure 8:**
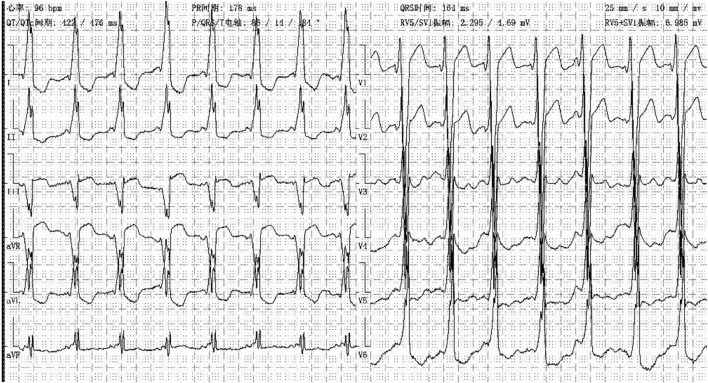
ECG showed WPW-B, left axis deviation in case one at her 6.5 years old.

**Figure 9:**
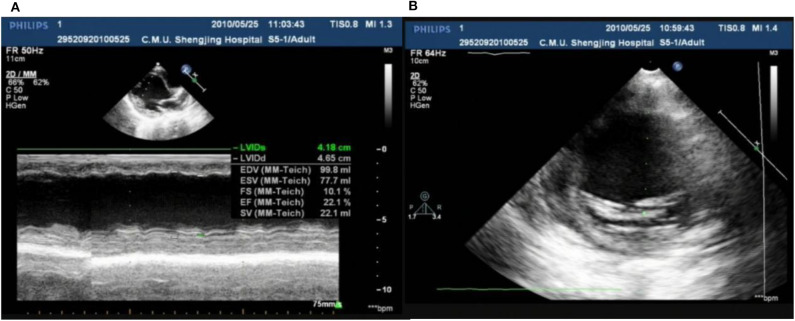
Echo showed LVED 46.5 mm, LVEF 0.22 **(A)**; endocardial thickness 3.5 mm **(B)** in case one at her 2 years old.

In our center, there were two cases of neonatal onset of SVT, presenting with normal QRS complex, increased LVED, and nearly normal cardiac function at the initial diagnosis. Three WPW cases were enrolled in our study, one (Case 1) (Figure 8) merits further description. She did not undergo radiofrequency ablation upon WPW diagnosis, and had significant cardiac dysfunction (LVEF = 0.22) (Figures 9A,B) in 2010. At that time (when she was 18 months of age), there was a lack of understanding of WPW associated cardiomyopathy and few hospitals in China carry out radiofrequency ablation for infants. It was commonly accepted that radiofrequency ablation in young children was associated with a risk of valve damage (20). We did not discuss fully radiofrequency ablation with her parents, and she was not followed up after discharge for economic reasons until her worsened cardiac insufficiency at 6 years of age (LVEF = 0.18). At this point, we explained the prognosis as well as the necessity and possible risks of radiofrequency ablation to her parents. Her parents decided on conservative treatment instead. She died at the age of 8 years, more than 6 years after her initial examination.

The prognosis of cardiomyopathy in children is related to arrhythmia. It has been reported that there are two age peaks in the symptoms of syncope or heart failure due to tachyarrhythmia in patients who have not had cardiomyopathy previously: within 1 year of age, and between 14 and 16 years of age. Syncope or heart failure due to tachyarrhythmia occurs in 80% of patients under 1 year of age (4). This phenomenon may be related to the repeated SVT or persistent SVT at a young age without presentation. Two elder DCM (8 and 11 years) patients with AT all died after diagnosis at 6 and 13 months, respectively. Both lived in countryside. Due to economic reasons, they did not see a doctor until they developed serious heart failures. Patients treated early were all recovered within 1 year.

Cardiomyopathy can casue cardiomyocyte remodeling and disarray (similar to that seen in hearts with scar formation) remodeled heart may cause recurrent PVC and VT which, in turn, leads to a decrease in cardiac function (1), resulting in difficult recovery. Based on our experience, low-dose digoxin (one-tenth saturation to one-eighth saturation; p.o.) can improve cardiac function even in the presence of frequent short-term paroxysmal VT, and LVEF can recover significantly.

If IVB occurs, myocardium contracts out of synchronicity, making it difficult to control heart failure. The ventricular septum is activated by the left ventricle under physiologic conditions. CLBBB completely changes the electrical activity of the left ventricle, and its activation originates from the right side. The electrical impulse travels down, to the left, and slightly forward. This leads to non-uniform and delayed depolarization of the left ventricle. In the presence of effective distal branches of the left beam and Purkinje neural network, the left ventricle can only work partially (21), leading to severely impaired cardiac function. Although the electrical activity in WPW-B, IVB and CLBBB is inconsistent, the mechanical activity of hearts in these three is similar. The electrical activity is first conducted through the bypass into the right ventricle, causing local myocardial excitation. However, conduction in myocardial cells is slow. In normal situation, electrical activity of sinoatrial node is slow traveling in HIS system, but becomes fast in Purkinje neural network after it passes HIS. This conduction along with the electrical activity traveling to the right ventricle completes the contraction of the left ventricle and part of the right ventricle. It is also a typical ventricular asynchrony. Therefore, we categorize them in one group. Cardiac resynchronization therapy has been shown to be efficacious to improve functional status and prolong the survival rate of patients with advanced chronic heart failure (22).

**Figure 10:**
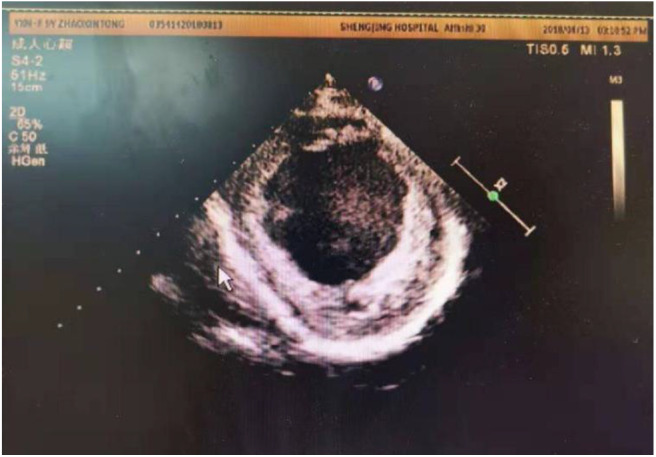
Echo showed LVED 64 mm, LVEF 0.24, endocardial thickness 3–5 mm in a 10 years old patient (about 6 years after pacemaker installed).

At present, criteria for implantation of a permanent pacemaker for CLBBB in children is lacking. If the criteria used in adults is adopted, some children will fail to meet them, but such criteria in children are needed urgently. Hence, specific guidelines for implantation of a permanent pacemaker in children must be based on scientific investigations, including pediatric clinical trials (23). Left-ventricular implantation of a permanent cardiac pacemaker in adults with CLBBB has shown good long-term results, and can improve LVED, LVEF and quality of life (24). In our center, a girl (Case 2) with EFE and CLBBB received a permanent pacemaker implantation. However, her cardiac function did not recover significantly (Figure 10), possibly due to problems in the right ventricle (25).

## Conclusions

The long-term prognosis of pediatric patients with cardiomyopathy is associated with the type of arrhythmia and time of intervention. The earlier arrhythmia is under control, the better the prognosis is. Patients with pre-excitation syndrome type-B, especially a significantly widen QRS complex, carry poor prognosis if radiofrequency ablation is not undertaken.

## Data Availability Statement

All datasets generated for this study are included in the article/Supplementary Material.

## Ethics Statement

The studies involving human participants were reviewed and approved by the Ethics Committee of Shengjing Hospital. Written informed consent to participate in this study was provided by the participants' legal guardian/next of kin.

## Author Contributions

HW: control all cases diagnosis, treatment, main idea, and data analysis. Y-QC: cases collection, data analysis, and case observation. X-YY: soma cases diagnosis and treatment. RC, Y-LX, X-XY, CW, LS, Y-MX, X-ML, and X-ZC: case observation.

## Conflict of Interest

The authors declare that the research was conducted in the absence of any commercial or financial relationships that could be construed as a potential conflict of interest.
